# Quantum healthy longevity from cells to cities

**DOI:** 10.3389/fragi.2024.1416447

**Published:** 2024-08-13

**Authors:** Tina Woods, Nic Palmarini, Lynne Corner, Richard Siow

**Affiliations:** ^1^ Collider Health, London, United Kingdom; ^2^ National Innovation Centre for Ageing, University of Newcastle, London, United Kingdom; ^3^ Ageing Research at King’s (ARK) and School of Cardiovascular and Metabolic Medicine and Sciences, Faculty of Life Sciences and Medicine, King’s College London, London, United Kingdom; ^4^ Department of Physiology, Anatomy and Genetics, Medical Sciences Division, University of Oxford, London, United Kingdom

**Keywords:** healthy longevity, exposome, resilience, healthspan, biomarkers

## Introduction

The general trend has been that we’re living longer–but life expectancy is falling and the gap in healthy life expectancy is widening between richest and poorest–in the richest countries in the world (GBD 2021 Demographics Collaborators, 2024).

The USA-the world’s richest country-is facing declining life expectancy despite investing more of its GDP in healthcare than any other country in the world (U.S. life expectancy at birth is three years lower than the OECD average, 2022). The UK’s economy meanwhile is being crippled by poor health, with workforce inactivity and lacklustre productivity gripping the government’s current attention.

### System change towards prevention

We have a broken “sickcare” model in the Western world and the burning platform to change things is growing quickly. System change is needed to focus on health as an asset to invest in, not as a cost in an increasingly unsustainable healthcare system buckling under the burden of poor health driven by growing health inequalities and aging populations.

The *Quantum Healthy Longevity Innovation Mission* was launched to address this and support the UK government’s manifesto commitment to extend healthy life expectancy while minimizing health and wellbeing inequalities. (Report et al., 2021).

The mission aims to harness frontier technologies including AI, quantum computing and systems biology to tackle big, real-world problems in climate and health (Woods et al., 2022), while understanding people’s needs and aspirations that contribute to maintaining health across the full life course (Ferrucci, 2020). This embraces the emerging research in computational approaches to the psychology of aging that links with biological and biomedical aging as well as the behavioural aspects for aging well, including nurturing brain health and investing in brain capital (Eyre et al., 2022).

## Focus on the exposome

**FIGURE 1:**
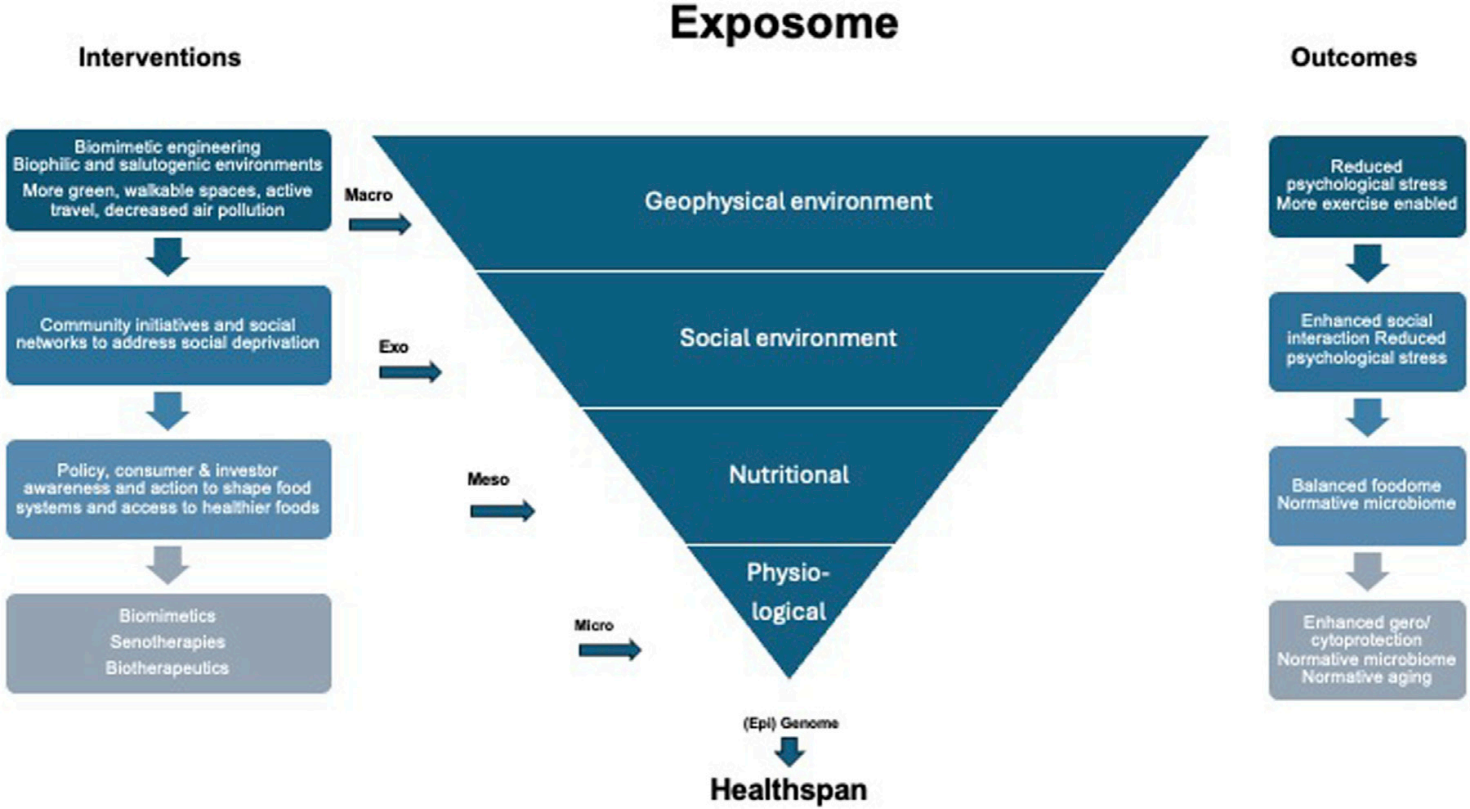
The Exposome and Health-Span. The different levels of interaction between the exposome with the (epi-)genome are shown with potential interventions to mitigate the effects of cumulative impact on health-span. The effects of the exposome from macro-geophysical environmental level to the micro-tissue and cellular physiology level and the potential outcomes are listed next to the respective levels. Specific exposome factors can be modified inspired by biomimetics. Design of urban environments to be more salutogenic, may be one aspect of the exposome that can be modified. Salutogenic effects can be driven by biophilia, an innate tendency to connect with natural environments, features of which have been evolutionary benefit. Adapted from Shiels et al. (2018).

We need to understand environmental factors encompassed in the exposome (Wild, 2005) in terms of helping humans not only to survive but to have the resilience to adapt to stress and to thrive in their “real world.” The “exposome” reflects the complex exposures humans face, which can lead to systemic chronic inflammation affecting lifelong health (Furman et al., 2019). The exposome includes the food we ingest, the air we breathe, the objects we touch, the psychological stresses we face, and the activities in which we engage, as detailed in Figure 1 (Shiels et al., 2021).

We also know that some people are more resilient than others when confronted with stress in their environment (Dantzer et al., 2018). Resilience is a concept getting more attention and relates to understanding how humans adapt to adverse conditions and recover from them, and reflects individual qualities such as personal control, a sense of purpose, and optimism (Liu et al., 2018).

### Psychological aging needs more attention

It is clear that human psychology is complex and changing behaviour difficult. We need to make it easy to keep healthy and well and cheap. We need the right environments to make the best choices. The simple fact is that if it is not easy or cheap or woven into the fabric of our daily lives, hopes and dreams it will not happen.

Sometimes it is the simple hope that a quick fix will work. More than half of adults in the USA take dietary supplements, yet most of the 90,000 supplements on the market have no evidence whatsoever that they improve health (Jia et al., 2022).

What many companies are selling are quick-fix hopes, not necessarily solutions that work. Hope is reflected in the placebo effect of course too-some say it will add 2 years to your healthy life expectancy. And despite all the promise of new longevity therapeutics, there is still no single drug or molecule yet available that can outdo the effects of a bad lifestyle or being in the poverty trap-if you do not eat healthily and you do not sleep or exercise enough or are stressed through financial worry and loss of hope, 10 years or more of your life is at risk.

Psychological aging is under-explored in research–yet connecting health and mind seems to be the focus of most of the longevity clinics emerging around the world treating real human beings. More research is needed to build on existing knowledge. For example, recent research analysing data from almost 12,000 adults showed psychological factors substantially contribute to biological aging; the pace of aging detected with a novel aging clock was shown to accelerate if a person feels unhappy, lonely, or has trouble sleeping (Galkin et al., 2022).

Also, older adults are exposed to additional psychological stressors such as bereavement and caregiving highlighting the importance of addressing social deprivation in the ageing population with such interventions as better social support networks.

All this points to the need for solutions that work not only on the biology of aging but the psychology of aging too, and also overall resilience, the ability of the body to adapt to stress. We need solutions which give people hope and allows them to follow their purpose, their reason for keeping alive. We also need solutions that are easy and accessible to everyone. Health needs to embrace the revolutions that have already come to other aspects in our lives, like banking and shopping–we are used to convenience and choice, and ever more so.

### Role of aging biomarkers

The latest developments are increasingly telling us we need to move upstream in health prevention. There has been a huge wave of biology breakthroughs spanning “omics” research and the “hallmarks of aging” as well as a better understanding of biological systems on the whole (López-Otín et al., 2013; Le Couteur and Barzilai, 2022).

In parallel of course we have seen an explosion in analytical and modelling technologies. Thanks to AI, we are getting increasingly powerful tools. DeepMind, for example, won the Breakthrough Prize in 2022 for its AlphaFold tool that has successfully predicted the structure of nearly all proteins known to science, but this was quickly overtaken by the latest AI kid on the block with generative AI that describes algorithms such as Chat GPT that can be used to create new content (including audio, code, images, text, simulations and videos).

Health data is plentiful, especially since most data we generate in our lives will affect or relate to our health in some way. This is gold stuff for biomarkers in aging research. Smartphone apps and wearable devices provide a largely untapped source of data about health behaviours in people’s day-to-day environments.

The Humanity app, for example, captures fitness and other cardio data from smart phones (such as Apple health data), sleep data from wearables (like the Oura ring); the app also captures and measures psychological markers (such as how you feel, whether you meditate, spend time in nature and are socially connected) and can upload blood tests results to analyze blood biomarkers. Taken together these markers captured digitally provide the capability for the app to analyse a range of biomarkers to give biological age assessments, which can then be used to track and monitor interventions.

GeroSense is a free research app that can calculate biological age from steps and heart rate data from your phone or wearable devices. The data from such apps capturing “digital biomarkers” are large in scale, can be collected at low cost, and often recorded in an automatic fashion, providing a powerful complement to traditional surveillance studies and controlled trials.

As the field advances, more sources of data like genetic, epigenetic, environmental and other “exposomic” data can be captured, shared and linked, yielding fuller analyses of individual health trajectories and providing more tailored health recommendations, while being able to be extrapolated at scale for population health measurement and design of future intervention trials.

While such apps can drive consumer behaviour, a prime example of citizen health engagement in the UK is Our Future Health (Our future health, 2024), that has been set up recently to recruit five million healthy volunteers in a major health study which aims to predict who will get ill in their later years even before they show symptoms to come up with new ways to prevent, detect and treat diseases such as dementia, cancer, diabetes, heart disease and stroke.

### Pro-innovation regulatory reform

The UK government is committed to leveraging “pro-innovation” regulatory reform, to unlock the potential of exponential advances we are seeing in science and technology (Science and Technology Framework, 2023) The legislation such as the Digital Information and Smart Data Bill that promotes data as an enabler, including use of personal data wallets and digital identity, will improve access for researchers and analysts to citizen-consented data which can link to the so-called world’s largest longitudinal ‘cradle to grave dataset’ from NHS healthcare records (Plan fo r Digital Regulation: developing an Outcomes Monitoring Framework 2022, 2023; Data saves lives: reshaping health and social care with data, 2022).

The UK Life Sciences Vision (Life sciences vision, 2021) outlines seven critical healthcare challenges that government, industry, the NHS, academia and medical research charities can work together on at speed to solve–from addressing aging to tackling dementia. This was the first time that research in the biology of aging was deemed a priority, and is one of the 7 “challenges.” The new government will consider how the Life Sciences Strategy may evolve in light of the intention to link health with economic growth in a long-term preventative health approach.

## Conclusion

Exploring the role of biomarkers to accelerate aging research is vital. Biomarkers could accelerate and shorten clinical trial times by acting as surrogate endpoints upstream in the health trajectory, and measure risk and progression of major age-related diseases. However, there is no agreed standard or list of approved aging biomarkers for clinical trials; and there is no international consensus or set of validated biomarkers of aging or aging clocks.

Most aging biomarkers considered to date are body- or performance-related (blood-based biomarkers, loss of functions, epigenetic clocks) while we know that psychological and emotional factors (often seen as soft, subjective metrics but which can now be collected by effortless data capture and measured as digital markers enabled with AI) have a major impact on healthspan and resilience. Anywhere from between 70%–90% of our health trajectory is defined by our lifestyle and exposome, and rarely measured and factored in even the most advanced medical practices.

We therefore need to go beyond biological and digital markers and explore “exposomic” biomarkers - understanding and measuring environmental markers of aging - bridging the biomedical perspective with the wider social, behavioural and environments angle and designing interventions to encompass all aspects of human flourishing taking a “cells to cities” approach.

This comes at a time where the need is growing to spread best practice and expertise, through a shared language backed up by standards, protocols and guidelines, to support and guide the growing number of longevity clinics, health optimisation centers, wellness retreats and indeed longevity-enabled cities of the future (as envisioned by the City of Longevity (City of Longevity: a new paradigm for cities in a longevity society, 2023) program at National Innovation Centre for Ageing), while minimizing reputational risk to the longevity sector through unscrupulous practice and poor science.

If we get it right and harness the incredible developments we are seeing with longevity science and ageing research, we can manage the demands of increasingly aging populations while extending healthspan for billions while protecting the planet.
